# The Impact and Functional Outcomes of Achilles Tendon Pathology in National Basketball Association Players

**DOI:** 10.4172/2329-910X.1000205

**Published:** 2016-09-23

**Authors:** Nirav H Amin, Kirk C McCullough, Gavin L Mills, Morgan H Jones, Douglas L Cerynik, James Rosneck, Richard D Parker

**Affiliations:** 1 Loma Linda University, Loma Linda, CA, United States; 2 Orthopaedic, Surgery, University of, Missouri, Kansas City, United States; 3 Loma Linda University, School of Medicine, Loma Linda, CA, United States; 4 The Cleveland Clinic Foundation, Euclid Avenue Cleveland, OH, United States; 5 Philadelphia Foundation for Orthopaedic Advancement, Philadelphia, PA, United States

## Abstract

Achilles tendon rupture within professional athletes has been shown to lead to devastating consequences regarding return to athletic performance. Not only can this devastating injury affect performance for the remainder of player's career, it frequently becomes a career-ending event. Considering these significant risks associated with complete rupture, the purpose of this study was to evaluate NBA players with a spectrum of reported Achilles tendon pathology, from tendinopathy (insertional and non-insertional) to complete rupture. Between the 1988-1989 and 2010-2011 NBA seasons, we identified 43 cases of Achilles tendon pathology treated non-operatively. A control group was matched for the players able to return to play with the following parameters: age, position played, number of seasons played in the league, and similarly rated career performance statistics. Considering the medical staff, trainers and facilities available to a professional athlete, a “weekend warrior” should be counseled that even in optimal conditions, 14% of NBA players were unable to return to function/play after Achilles tendinopathy, and that those who were able to return did so at a decreased level of performance. In conclusion, players with Achilles tendinopathy have a better chance to return if they are younger in age and early in their professional career. Furthermore, the association between Achilles pathology and decline in player performance is an important message to convey to coaching staff and team management to allow properly informed decisions when these conditions arise.

## Introduction

Basketball is a very dynamic sport, which places significant demands on the allowable physiologic stresses of tendons and ligaments of the lower extremities. It is all too common that these excessive demands lead to pathologic conditions after prolonged basketball play exposure. Although a significant proportion of reported foot and ankle injuries in basketball involve the lateral ankle ligaments the Achilles tendon plays a critical role within the sport-specific demands of basketball play [1-4]. It is frequently subjected to a significant amount of strain and eccentric loading due to its critical function in player acceleration, particularly with sudden stops and changes in direction when the foot is dorsiflexed and knee is extended. Risk factors associated with Achilles injuries include athletes of older age, changes in player performance characteristics, altered foot/ankle biomechanics, and anatomic variations [5,6]. Achilles tendon rupture within professional athletes has been shown to lead to devastating consequences regarding return to athletic performance [7,8]. This devastating injury not only affects performance for the remainder of player's career, but also frequently becomes a career-ending event. Considering the significant risks associated with complete rupture, the purpose of this study was to evaluate NBA players with a spectrum of reported Achilles tendon pathology, from tendinopathy (insertional and non-insertional) to complete rupture. Although, clinically not as significant as a complete rupture, it is critical to understand the negative effects on the performance of those who return to play to help guide NBA front offices, medical and coaching staffs on expectations of the individual player.

## Materials and Methods

Between the 1988-89 and 2010-11 NBA seasons, we identified 43 cases of Achilles tendon pathology treated non-operatively (Group 1 (Tendinopathy)). These cases included various types of insertional and non-insertional tendinopathy (e.g., tendinitis, paratendinitis, tendinosis/partial-thickness tearing). Players with Achilles pathology were identified through injury reports, press releases, and player profiles available to the public. A control group was matched for players returning to competition using the following parameters: age, position played, number of seasons played in the league, and similarly rated career performance statistics. Prior to data collection, a power analysis was conducted using an independent-samples test to evaluate the hypothesis that Group 1 (Tendinopathy) cases differed from controls. A two-sided t test with a significance level of 0.10 and a power of 0.80 was used to detect a moderate effect size of 0.60, with a standard deviation of 1. This analysis resulted in a required sample size of 23 players in each group. Our study identified 35 controls for 43 cases, and as such exceeded these statistical requirements. In addition to the aforementioned Group 1 (Tendinopathy) comparison with matched controls, we also compared Group 1 (Tendinopathy) with a group of NBA players (n=18) with reported complete rupture of the tendon treated by surgery during the same time period (Group 2 (Rupture)).

Demographic statistics were calculated for age, height, body mass index (BMI), experience (years played in the league), and other characteristics of the group. Players within Group 1 (Tendinopathy) who returned for at least 1 season (n=36) were compared using a two-tailed paired t test to determine the statistical significance in each player's performance before and after injury. To compare participants in Group 1 (Tendinopathy) with their matched controls, we used independent-samples t tests. Variables considered in this analysis included age, height, BMI, number of years prior to injury or index year, player position, Player Efficiency Rating (PER), and number of minutes played per game 2 years before injury or index year. PER is an assessment system commonly used to evaluate NBA players' performance and is calculated following the previously published formula [7]. The formula is as follows: ([points + rebounds + assists + steals + blocks] – [{field goals attempted – fields goals made} + {free throws attempted – free throws made}] + turnovers) / games. To determine whether a player returned for a “meaningful” career after injury (defined as returning for at least one season post injury), we used both univariate and multivariate logistic regression models. Continuous variables like age and BMI were also included in separate models where they were treated as categorical variables (age>30 years; BMI>25). Player positions were considered categorically as centers, forwards, or guards, with forwards used as the reference group because there were a large number of them in the study. Statistical analysis were performed using SAS version 9.3 (SAS Institute, Cary, North Carolina).

## Results

Thirty-seven of the 43 players (86%) in Group 1 (Tendinopathy) returned to play for at least one season after the injury, while six never returned to the NBA. Table 1 shows the average age, height, BMI, and experience for all players in the Group 1 (Tendinopathy).

The average age of players was 28.8 years, with a range of 22 to 39 years of age. For players who returned for at least one season, the average age was 28.3. Considering the performance of these returning players two years prior to injury, the average PER was 10.9 and the average number of minutes played was 23.6. Players that returned for at least one season, on average, played greater minutes per game (24.0) than those who did not return (21.5).

However, the players that did not return were on average older (32.3 years) and had more seasons in the league prior to reported Achilles injury/pathology (9.8 seasons).

Table 2 displays the characteristics of Group 1 (Tendinopathy) and their controls in terms of means and counts. Between the two groups, there were a total of 13 centers, 28 forwards, and 29 guards. Controls were appropriately matched to cases with respect to age, height, BMI, performance, experience and position, as indicated in this table by the various tests performed.

Performance variables (PER and number of minutes played per game) for Group 1 (Tendinopathy) players who returned to the NBA for at least one season after injury significantly declined post-injury as compared to their pre-injury variables as shown in Table 3. The mean difference in PER for those that returned for at least one season was significant (1.27, p=0.06). The number of minutes played per game also declined, where the mean difference was significant (2.53, p=0.02). Table 4 shows the comparison between Group 1 (Tendinopathy) and controls in terms of performance variables before and after injury or index year. Although the controls actually had a greater mean decline in performance than those with Achilles tendinopathy, these performance variables were not statistically different between the two groups.

To validate our statistical analysis, we first examined each univariate binary logistic regression model for all possible confounders to influence return to play after injury (Table 5). The variables of age and number of pre-injury seasons were statistically significant at the nominal level of 0.10. We then evaluated the correlation of all possible confounders (Table 6), and noted that the performance variables were significantly correlated with several predictors (Table 7) positive correlations between minutes played per game two years before injury and age (ρ=0.29), pre-injury years (ρ=0.39), and PER two years before injury (ρ=0.91).

Table 7 shows the comparison of performance variables before and after the injury/index year. While there appears to be no large differences between cases and controls with respect to field goals and free throws, the steals per game appear to decline more after index year for the controls (although not statistically significant). When considering several performance variables per 40 minutes of play, there appears to be larger declines for the controls in comparison to the cases, although not statistically significant.

After conducting the univariate analyses and considering the correlation among potential predictors that affect return to competition, we also considered a multivariate logistic regression model. Using a forward selection approach, our final model included both number of pre-injury years and minutes played per game two years before injury. Because of the significant correlation between these two predictors, and to reduce multicollinearity, we only focused on the univariate results presented in Table 5. Therefore, the odds of returning after an injury were significantly decreased by 21% for every 1-unit increase in the number of years played prior to injury (Table 5: OR=0.79, p=0.027). Similarly, the odds of returning were significantly decreased by 14% for every 1-unit increase in age (Table 5: OR=0.86, p=0.089). In summary, players with more seasons played and those older in age at time of reported Achilles pathology are less likely to return to competition after injury.

Comparing Group 1 (Tendinopathy) with Group 2 (Rupture), we found no differences in their demographics or performances prior to injury except that Group 2 (Rupture) had more years in the league than Group 1 (Tendinopathy) (Table 8, p=0.08). The players in both groups who returned to competition had declines in performance and consistently played less minutes in games upon returning (Table 9). Despite these similarities, we noted a difference in the pre- to post-injury PER comparison between Group 1 (Tendinopathy) and 2 (Rupture) players, where players in Group 2 (Rupture) have a statistically significant larger difference in PER statistics compared to the players in Group 1 (Tendinopathy) (Table 10, p=0.036). Furthermore, although there is a noticeable difference in minutes played per game between players in the two groups, this difference was not found to be statistically significant.

Table 11 shows the comparison of performance variables before and after the injury/index year. With the exception of free throws, there appears to be a significant difference in all performance variables between Group 1 (Tendinopathy) and 2 (Rupture) players with larger declines post-injury for players in Group 2 (Rupture) compared to Group 1 (Tendinopathy).

## Discussion

While inherently not as functionally and clinically significant as a complete rupture, this study demonstrates that Achilles tendinopathy remains a significant problem related to performance measures. To our knowledge, this is the first study within a cohort of active professional basketball athletes that reports on the entire spectrum of Achilles pathology and its effect on sport participation dynamics.

Considering the physical demands of competing at the professional level, we hypothesized that NBA player performance metrics in patients with Achilles tendinopathy would be negatively affected. In the cohort group (Group 1(Tendinopathy)) with Achilles tendinopathy, players played less minutes (p=0.023) and exhibited decreased PER metrics (p=0.061), following the trend demonstrated by Amin et al. in those with complete ruptures [7].

In their 2013 publication, Gajhede-Knudsen et al. reported on the largest series of Achilles tendon disorders over an 11-year period in professional soccer [9]. In their study cohort, players missed an average of 23 days for Achilles tendinopathy, exhibited a higher symptomatic recurrence rate after shorter recovery periods, and were more commonly within the older cohort of active players (average age of 27.2 years). This negative effect of player age is consistent with our current findings which demonstrate the odds of returning being significantly decreased by 14% for every 1-unit increase in age (Table 5; OR=0.86, p=0.089).

In addition to player-specific performance dynamics, the effects of player injury on overall team performance are important considerations for coaches and professional team management. Eirale et al. studied the relationship with between individual injuries and overall performances by the team, demonstrating that lower injury incidence was positively correlated with team success [10]. Using the injury burden as total days lost from injury and evaluating effects of injury on team performance, Hagglund et al. also demonstrated the negative impact of player injuries on overall team performance [11]. Therefore, while players are often eager to return to play as soon as possible, it is important to ensure that those with Achilles tendon pathology are given appropriate recovery periods before returning to competitive play not only for a reduced risk of re-injury as shown by Gajhede-Knudsen et al. [9] but also to benefit team performance in the short and long term.

While there is an abundance of medical literature documenting the frequency of injuries within various sport populations, limited literature exists regarding the effect of injury severity on player performance. Amin et al. published a series of complete ruptures in NBA players and found 7 out of 18 players were unable to resume their career after a complete Achilles rupture [7]. In their study on Achilles rupture in the NFL, Parekh et al. found 10 out of 31 NFL players were unable to resume their career after a complete rupture [8]. Utilizing this data, it is imperative to educate the medical staff, players, and front office personal regarding the performance matrix based on injuries. It also allows physicians to counsel athletes about setting realistic expectations about their ability to return to prior performance levels [7,8]. Considering the medical staff, trainers and facilities available to a professional athlete, a “weekend warrior” should be counseled that even in optimal conditions, 14% of NBA players were unable to return to function/play after Achilles tendinopathy, and that those who were able to return did so at a decreased level of performance.

Although some have proposed that untreated Achilles tendinopathy could predispose athletes to rupture, we were only able to identify one case of reported Achilles tendinopathy that progressed to complete rupture necessitating surgery within our retrospective cohort of NBA athletes from Group 1 (Achilles tendinopathy) to Group 2 (complete Achilles rupture). Considering the demands of a player, a further study is needed with a larger subset of patients to more definitively evaluate this potential relationship and implications associated with continued stress of basketball play on the Achilles tendon.

One strength of the current study was using a standard league– specific performance measure (PER) that has been previously utilized in the medical literature to assess the outcomes of the players [7]. This effectively eliminates any potential bias that would be produced if we had designed an improvised tool for measuring the performance outcome. Furthermore, the utilization of retrospective reviews of clinical pathology and statistical performance measures captured from online, web-based sources has been largely accepted in the literature as an effective and accepted means of reporting on elite athletes in situations where access to clinical data is restricted [1,7,8,12,13].

This study presents a few limitations, however, including the inability to reliably correlate each specific non-rupture Achilles pathology (e.g., insertional/non-insertional tendinopathy) to the reported outcome. However, our primary goal was to determine the level of change in performance when Achilles tendon pathology occurred compared to the control group. This study clearly establishes that complete rupture is not the only clinically concerning entity of the Achilles as it relates to functional performance. Additional prospective studies aimed at specific diagnoses of Achilles tendinopathy should be performed to further analyze the effect of each specific condition on player performance. Other study limitations include the relatively small sample size and bias of a retrospective cohort design. As there is no publicly accessible comprehensive database that documents Achilles pathology in the NBA combined with long-term follow-up data of those who have undergone treatment, it is possible that not all NBA athletes with Achilles tendinopathy were included in our study. However, as our current methodology has been similarly utilized in multiple studies in the medical literature [7,8,12-14]. We believe that our study sample is representative of the NBA athletes who suffer from these conditions and is the best option to study clinical outcomes in these players at the current time.

## Conclusion

In conclusion, players with Achilles tendinopathy have a better chance to return to competition if they are younger in age and early in their professional career. Furthermore, the association between Achilles pathology and decline in player performance is an important message to convey to the coaching staff and team management so that informed personnel decisions can be made. On a broader scale, these findings also help to provide expectations for the “weekend warrior” with Achilles pathology regarding their ability to return to activity.

## Figures and Tables

**Table 1:** Demographic and performance data for study group 1^a^.

Variable	All	Returned for >1 Season	Did Not Return
Age, y	28.8 (22.0, 39.0)	28.3 (22.0, 39.0)	32.3 (24.0, 39.0)
Height, cm	200.6 (180.0, 221.0)	200.3 (180.0, 221.0)	202.5 (191.0, 216.0)
BMI	25.4 (22.0, 31.5)	25.3 (22.0, 30.3)	25.9 (22.0, 31.5)
Pre-injury seasons in NBA	6.0 (0.0, 18.0)	5.4 (0.0, 13.0)	9.8 (2.0, 18.0)
PER 2 Years before injury	10.9 (1.1, 24.3)*	11.2 (1.1, 24.3)*	9.2 (4.6, 17.7)
Minutes played per game 2 years before injury	23.6 (4.7, 39.4)*	24.0 (4.7, 39.4)*	21.5 (11.3, 30.7)

a Values are expressed as mean (minimum, maximum). BMI: Body Mass Index; NBA: National Basketball Association; PER: Player Efficiency Rating.

* missing three observations

**Table 2:** Demographic and performance comparison between group 1 and controls a.

Variable	Case	Control	P Value
Age, y	28.5	27.8	0.497^b^
Height, cm	199.7	199.5	0.907^b^
BMI	25.3	24.6	0.125^b^
Pre-injury/index Seasons	5.5	5.5	0.973^b^
PER 2 years before injury/ index	11.3	12.3	0.495^b^
Minutes played per game 2 years before injury	24.3	25.7	0.519^b^
Position, n			0.946^c^
Center	7	6	
Forward	14	14	
Guard	14	15	

a Values are express as the mean unless otherwise indicated. BMI: Body Mass Index; NBA: National Basketball Association; PER: Player Efficiency Rating.

b Performed with independent-samples t test, equal variances not assumed.

c Performed with test.

**Table 3:** Difference in performance variables between preinjury and postinjury seasons in NBA Players in group 1 a.

Variable	Decline in Players Who Returned for At Least 1 Season After Injury	P value
Minutes played per game	2.53 (0.38 to 4.68)	0.023^b^
PER	1.27 (-0.06 to 2.61)	0.061^b^

a Values are expressed as mean (95% confidence interval). The difference in mean performance variables between 2 years before injury and at least 1 season after injury year was assessed for each variable using paired-samples t tests between seasons. Mean differences per player are represented as positive or negative, where positive differences indicate greater numbers preinjury versus postinjury. Includes only players who returned for meaningful careers (>1 season, n = 34). NBA, National Basketball Association; PER: Player Efficiency Rating.

b Statistically significant at.

**Table 4:** Comparison between NBA players performance in group1 and controls a.

Variable	Cases (Pre-injury – Post-injury)	Controls (Pre-index – Post-index)	P value
Minutes played per game	2.53	3.82	0.400
PER	1.27	2.63	0.125

a Comparison between cases and controls performed with independent-samples t tests, equal variances not assumed. NBA: National Basketball Association; PER: Player Efficiency Rating.

**Table 5:** Univariate binary logistic regression for variables that possibly influence return to play after injury in group 1^a^.

Variable	Odds Ratio (95% CI)	P value
BMI (continuous)	0.88 (0.59, 1.31)	0.525
BMI (> 25)	1.70 (0.28, 10.45)	0.567
Height	0.98 (0.90, 1.07)	0.626
Age (continuous)	0.86 (0.73, 1.02)	0.089^b^
Age (> 30 yrs)	0.21 (0.03, 1.33)	0.098^b^
Center	0.55 (0.09, 3.58)	0.533
Guard	1.36 (0.22, 8.41)	0.738
Forward	1.22 (0.20, 7.53)	0.833
Pre-injury Seasons	0.79 (0.63, 0.97)	0.027^b^
PER 2 Years Before Injury	1.06 (0.90, 1.25)	0.470
Minutes played per game 2 years before injury	1.04 (0.94, 1.15)	0.512

a BMI: body mass index; CI: Confidence Interval; NBA: National Basketball Association; PER: Player Efficiency Rating. significant at.

**Table 6:** Correlation matrix for variables that possibly influence return to play after injury (group 1).

Variable	V1	V2	V3	V4	V5	V6
Age, y (V1)	1.00					
Height, cm (V2)	-0.34^b^	1.00				
BMI (V3)	-0.21	0.27^b^	1.00			
Pre-injury/index Seasons (V4)	0.70^b^	0.08	-0.03	1.00		
PER 2 years before injury/index (V5)	0.14	0.13	0.10	0.31^b^	1.00	
Minutes played per game 2 years before injury (V6)	0.29^b^	-0.10	-0.10	0.39^b^	0.91^b^	1.00

a Pearson correlation coeficient. Test of zero correlation between variables. BMI: Body Mass Index; CI: Confidence Interval; NBA: National Basketball Association; PER: Player Efficiency Rating.

b Statistically significant at.

**Table 7:** Comparison between NBA players in group 1 and controls regarding specific activities^a^.

Variable	Cases (Post-injury – Pre-injury)	Controls (Post-index – Pre-index)	P value
Field goals	-0.02	-0.01	0.603
Free throws	-0.01	0.01	0.329
Rebounds per game	-0.48	-0.93	0.211
Steals per game	0.00	-0.14	0.138
Blocks per game	-0.07	-0.13	0.520
Rebounds per 40 min	0.09	-0.22	0.261
Steals per 40 min	0.12	0.04	0.438
Blocks per 40 min	-0.00	-0.09	0.455

a Comparison between cases and controls performed with independent-samples t tests; equal ariances not assumed. NBA: National Basketball Association.

**Table 8:** Demographic and performance comparison between group 1 and 2^a^.

Variable	Group 1	Group 2	P Value
Age, y	28.8	29.7	0.399^b^
Height, cm	200.6	203.1	0.346^b^
BMI	25.4	25.6	0.719^b^
Pre-injury Seasons	6.0	7.6	0.080^b^
PER 2 years before injury/index	10.9	12.2	0.497^b^
Minutes played per game 2 years before injury	23.6	25.4	0.520^b^
Position, n			0.885^c^
Center	10	5	----------------
Forward	16	7	
Guard	17	6	

a Values are expressed as the mean unless otherwise indicated. BMI, body mass index; PER: Player Efficiency Rating.

b Performed with independent-samples t test, equal variances not assumed.

c Performed with test.

**Table 9:** Difference in performance variables between pre-injury and post-injury seasons in group 1 and 2^a^.

Variable	Decline in Players Who Returned for At Least 1 Season After Injury	P value
Group 1		
Minutes played per game	2.53 (0.38 to 4.68)	0.023^b^
PER	1.27 (-0.06 to 2.61)	0.061^b^
Group 2		
Minutes played per game	5.11 (0.99 to 9.23)	0.020^b^
PER	4.57 (1.72 to 7.42)	0.005^b^

a Values are expressed as mean (95% confidence interval). The difference in mean performance variables between two years before injury and at least one season after injury year was assessed for each variable using paired-samples t tests between seasons. Mean differences per player are represented as positive or negative, where positive differences indicate greater numbers pre-injury versus post-injury. Includes only players who returned for meaningful careers (>1 season). NBA: National Basketball Association; PER: Player Efficiency Rating.

b Statistically significant at.

**Table 10:** Comparison between group 1 and 2 in performance.

Variable	Partial (Pre-injury – Post-injury)	Complete (Pre-injury – Post-injury)	P value
Minutes played per game	2.53	5.11	0.243
PER	1.27	4.57	0.036^b^

a Comparison between the two groups performed with independent-samples t tests, equal variances not assumed. NBA: National Basketball Association; PER: Player Efficiency Rating.

b Statistically significant at.

**Table 11:** Comparison between group 1 and 2 regarding specific activities^a^.

Variable	Group 1 (Post-injury – Pre-injury)	Group 2 (Post-index – Pre-index)	P value
Field goals	-0.02	-0.05	0.017^b^
Free throws	0.02	-0.06	0.209
Rebounds per game	-0.45	-1.23	0.058^b^
Steals per game	-0.01	-0.23	0.015^b^
Blocks per game	-0.07	-0.37	0.014^b^
Rebounds per 40 min	0.10	-0.68	0.081^b^
Steals per 40 min	0.10	-0.12	0.074^b^
Blocks per 40 min	-0.02	-0.40	0.006^b^

a Comparison between Group 1 and Group 2 performed with independent-samples t tests; equal variances not assumed.

b Statistically significant at 0.10.
